# Experience in Transoral Robotic Surgery in Pediatric Subjects: A Systematic Literature Review

**DOI:** 10.3389/fsurg.2021.726739

**Published:** 2021-08-12

**Authors:** Matteo Vianini, Giacomo Fiacchini, Giacomo Benettini, Iacopo Dallan, Luca Bruschini

**Affiliations:** Otolaryngology, Audiology and Phoniatric Operative, Department of Surgical Pathology, Medical, Molecular and Critical Area, Azienda Ospedaliero-Universitaria Pisana, University of Pisa, Pisa, Italy

**Keywords:** pediatric surgery, pediatric trans-oral robotic surgery, pediatric TORS, pediatric Da Vinci, trans-oral robotic surgery

## Abstract

Pediatric transoral robotic surgery (TORS) has improved from 2007 to 2020, widening its indications and feasibility. This article aims to systematically analyze the procedures performed from the first use until the current year, observing their evolution over time. A systematic literature review was performed using PubMed, Scopus, Web of Science, and Cochrane databases between March 1, 2000, and April 1, 2020. We selected studies that were written only in English and were performed in live human subjects. About 16 studies were found with a total of 73 subjects treated, among them 41 were men and 32 were women with an average age of 6.8 ± 4.99 years. There have been four (5.47%) conversions. Both functional and benign-malignant diseases have been treated in the series. Eleven (15.06%) pre-operative tracheostomy and zero post-operative tracheostomy were performed. The bleeding data was only reported in 9 studies and was <50 ml. Only one (1.36%) intra-operative complication and 10 (12.32%) postoperative complications were reported. We consider the TORS procedures in pediatric subjects safe, feasible and with good surgical outcomes up to the laryngeal region.

## Introduction

Since 2000, when the Da Vinci robotic system (Intuitive Surgical Inc., Sunnyvale, CA, USA) was approved for laparoscopic surgery by the Food and Drug Administration (FDA), robotic surgery has been progressing not only in terms of technology but also in terms of possibilities for application.

In the beginning, the system was mainly used in abdominal surgery for urological and gynecological procedures. Successively, it was applied in other anatomical districts with exponential growth in the population of pediatrics. In 2009, the FDA approved the Da Vinci system for transoral procedures.

The purpose of this study is to systematically analyze the transoral robotic surgery (TORS) procedures performed in pediatric subjects, from the first cases reported (1), up until today, to observe the evolution of the procedure in terms of feasibility and prevalence of the anatomical sites treated.

## Materials and Methods

### Literature Review and Research Strategy

A systematic literature review was conducted using PubMed, Scopus, Web of Science, and Cochrane databases, according to the PRISMA guidelines (2), by combining the following keywords: robotic, robotic surgery, pediatric tors, children head and neck, Da Vinci, pediatric transoral robotic surgery, pediatric robot ENT, pediatric robot otolaryngology, tors infant, tors children; between March 1, 2007, and April 1, 2020.

### Eligibility Criteria

We included studies according to the following criteria: studies on TORS pediatric procedures; retrospective and prospective studies that are peer-reviewed; English-written studies; studies performed in live human subjects (cadaver dissections were excluded). The pediatric subject included was up to 16 years of age.

### Study Selection and Data Extraction

Two of the authors (MV and GF) independently screened the retrieved studies based on the title, keywords, and abstracts to exclude irrelevant and non-English written studies. Duplicates were removed and the full-text of the remaining papers were analyzed when uncertainty existed in the abstract evaluation. A manual search in the reference lists of these articles was performed to identify potentially relevant papers missed during the database search. Differing opinions were solved by consensus between the two authors. Data extracted and analyzed for the study included the following criteria: the demographic data, the number of procedures performed, the number and type of pathology, the typology of the robot used, site and sub-site of the procedure, surgical time (ST) of the procedures, number of conversions and/or abortions, the number of intra- and post-operative complications, times of hospitalization, intraoperative blood loss, ways for food intake, and the number of intra- and post-operative tracheostomy.

### Evidence Quality Appraisal

All studies were assessed for their methodological quality using the National Institute for Health and Care Excellence (NICE) methodology checklist (3) for quality assessment of case series.

### Statistical Analysis

The data from each study were transcribed in tabular forms and these were summarized using descriptive statistics. Dichotomous variables were reported as numbers and percentages, while continuous variables were reported as mean ± SD, or median ± interquartile range (IQR) if the values were not normally distributed.

## Results

**Figure 1:**
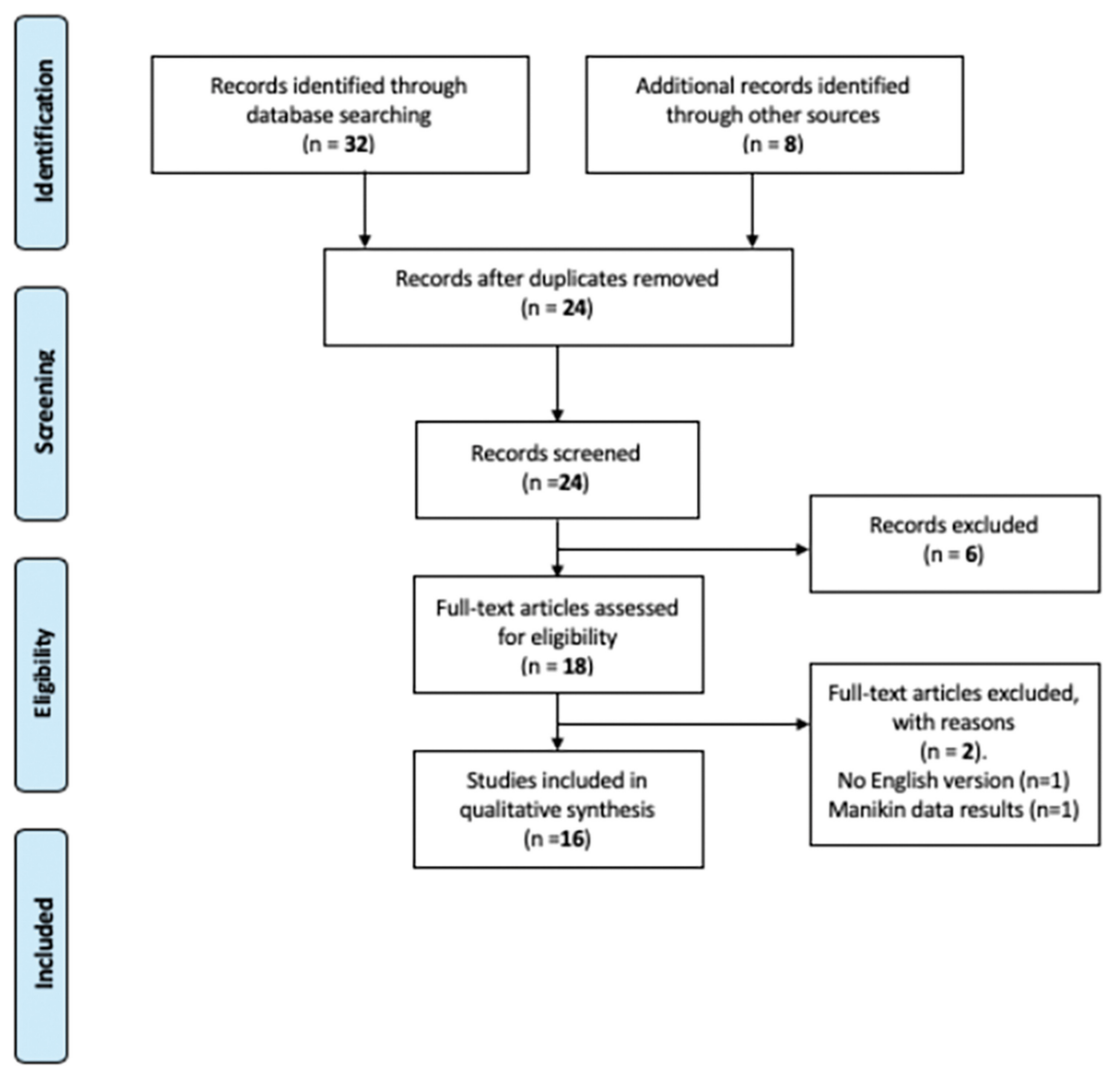
Prisma 2009 flow diagram for TORS procedure performed in pediatric subjects.

General data about included studies and subjects are shown in Table 1. Sixteen studies (Table 2) met our inclusion criteria (Figure 1).

**Table 1:** Summary table of the study outcomes.

**Data analyzed**	**Outcomes**
Retrospective	*n* = 8 (50%)
Case report	*n* = 8 (50%)
*N*. subjects	*n* = 73
Male	*n* = 41 (56.16%)
Female	*n* = 32 (43.83%)
Mean age	*n* = 6.8 (±4.99 SD) years old
Younger age	*n* = 14 days
Da Vinci Si	*n* = 69 (94.5%)
Da Vinci Xi	*n* = 4 (5.47%)
Conversion	*n* = 4 (5.47%)
Pre-operative tracheostomy	*n* = 11 (15.06%)
Post-operative tracheostomy	*n* = 0 (0%)
Bleeding <50 ml	*n* = 9
Bleeding n.a	*n* = 7
Food intake	*n* = 73
Post-operative oral diet	*n* = 44 (60.27%)
Nasogastric tube	*n* = 1 (1.36%)
Gastrostomy (2 preexisting)	*n* = 3 (4.10%)
Diet n.a	*n* = 25 (34.24%)
Hospitalization	*n* = 3.06 (±3.95 SD) days
Intra-operative complication	*n* = 1 (1.36%)
Post-operative complication	*n* = 10 (12.32%)

*n, numbers; SD, standard deviation*.

**Table 2:** Database.

**Pediatric TORS 2015–2020 **	**Study design**	**Number of patients (*n*) **	**M (*n*)**	**F (*n*)**	**Mean Age (*n* ± SD or range)**	**Age ofthe youngestpatient inthe study (*n*) **	**Pathology (*n*)**	**Localization (*n*)**	**Procedure (*n*)**	**Surg. Time (*n*)**	**Da Vinci Si (*n*)**	**Da VinciXi (*n*) **	**Conversion (*n*)**	**Tracheotomy/pre operative (*n*) **	**Tracheotomyi ntra/post operative (*n*) **	**Intraoperative Blood loss (*n*) **	**Oral diet (*n*) **	**Gastrostomy (*n*) **	**NGT (*n*) **	**Hospitalization days (*n* ± SD or range) **	**Intraoperative complications (*n*) **	**Postoperative complications (*n*) **
Rahbar et al. (1)	Retrospective	5	2	3	60 months	12 months	5: laryngeal cleft	5: larynx	2: laringeal cleft repair 3: no TORS but conversion	n.a	5	0	3	n.a	0	n.a	n.a	n.a	n.a	n.a	n.a	n.a
Kokot et al. (4)	Case report	1	0	1	180 months	180 months	1: oropharyngeal synovial sarcoma	1: oropharingeal wall	1: partial pharyngectomy and partial glossectomy	n.a	1	0	0	1	0	n.a	0	0	1	3 days	0	0
Wine et al. (5)	Case report	1	1	0	17 months	17 months	1: high-grade undifferentiated sarcoma	1: oropharynx (soft palate)	1: resection included the left hemipalate, left lateral oropharynx, 25% of left base of tongue, and the left lateral oropharynx, including the anterior and posterior pillars + FAMM	70 min	1	0	0	0	0	0	0	1	0	16 days	0	0
Kayhan et al. (6)	Case report	1	0	1	2 months	2 months	1: LTGDC	1: BOT	1: excision	3 min	1	0	0	0	0	n.a	1	0	0	3 days	0	0
Leonardis et al. (7)	Retrospective study	16	11	5	144 months	60 months	11: OSAS. 2: dysphagia. 1: upper airway obstruction. 1: recurrent tonsillitis 1: exercise-induced breathing difficulty	16: BOT	16: lingual tonsillectomy	34 min	16	0	0	2	0	5.9 mL (range 2–10)	14	2 preexisting	0	1 (range 2–13) day	0	2: post operative bleeding. 2: pneumonia.
Leonardis et al. (8)	Retrospective study	5	3	2	21.6 months	15 months	5: laryngeal cleft	5: larynx	5: laryngeal cleft repair	102 to 36 min	5	0	0	0	0	2.6 ml (range 2–5 ml)	5	0	0	4.2 days	1 minor buccal laceration.	0
Ferrell et al. (9)	Retrospective	3	2	1	96 months	36 months	1: posterior glottic stenosis 1: laryngeal cleft. 1: idiopathic bilateral. vocal cord paralysis.	3: larynx	1: posterior cricoid split with cartilage graft placement. 1: repair. 1: left posterior cordectomy and subtotal arytenoidectomy.	n.a	3	0	1	3	0	n.a	n.a	n.a	n.a	1 not reported data. 1: 0 days 1: 0 days	0	1: oral tongue edema, decreased oral intake, and suspected aspiration (5 days later).
Thottam et al. (10)	Retrospective	9	5	4	126 months	62 months	9: OSAS	9: BOT	9: Lingual tonsillectomy	n.a	9	0	0	0	0	0	9	0	0	5: 1 day 1: 3 days 1: 14 days 1: 12 days. 1: not reported data	0	1: post-operative bleed. 1: pneumonia.
Carroll et al. (11)	Case report	1	1	0	72 months	72 months	LTGDC	1:BOT	1: cut of tongue muscle with the mass. No hyoid bone resection	28 min	1	0	0	0	0	<5 ml	1	0	0	1 day	0	0
Zdanski et al. (12)	Retrospective case series	16	6	10	48 months	14 days	• 1: hamartoma. 1: Lymphatic malf. • 7: laryngeal cleft. • 2: saccular cyst. • 2: pharingo-esophageal strictures. 3: hypopharynx-supraglottis lynphatic malformation.	1: BOT. 1: BOT. 7: larynx. 2: larynx. 2: pharynx-esophagus. 3: hypopharynx-larynx.	1: resection. 1: excision. 7: repair. 2: removal. 2: release. 3: excision	2h 24 min	16	0	0	3	0	from 0 to 25 ml	n.a	n.a	n.a	from 1 to 20 days	0	1: pharingeal-esophageas stricture.
Montevecchi et al. (13)	Case report series	3	3	0	159 months	132 months	3: OSAS	3: BOT	1: TBR. 1: TBR + eoiglottoplasty 1: TBR + eoiglottoplasty + adenotonsillectomy	n.a	3	0	0	0	0	n.a	3	0	0	1: 5 days 1: 4 days 1: 4 days	0	0
Canevari et al. (14)	Case report	1	1	0	192 months	192 months	1: Ewing's sarcoma	1: BOT	1: partial glossectomy	n.a	1	0	0	0	0	n.a	1	0	0	2 days	0	0
Kayhan et al. (15)	Retrospective case series	8	4	4	66 months	1.5 months	4: LTGDC. 1: vallecular cyst. 1: lingual thyroid. 1: minor salivary. gland tumor. 1: bronchogenic cyst.	8: BOT	8: dissected completely and removed from the intact lingual muscle layer at the base of tongue	8.8 ± 6.9 min	7	1	0	1	0	1 <5. 1 <10. 1 <10. 1 <10. 1 <10. 1 <10. 1 <10. 1 <50	8	0	0	1: 1 day 1: 3 days 1: 1 day 1: 3 days 1: 1 day 1: 1 day 1: 4 day 1: 4 day	0	1: minor bleeding 10 days after surgery.
Arnold et al. (16)	Case report	1	0	1	72 months	72 months	1: neurofibroma	1: supraglottic extending laterally into the parapharyngeal and carotid space	1: removed mass	50 min	0	1	0	1	0	n.a	n.a	n.a	n.a	1	0	0
Turhan et al. (17)	Case report	1	1	0	3 months	3 months	1: LTGDC.	1: BOT	1: excision.	10 min	0	1	0	0	0	0	1	0	0	7 days	n.a	0
Venkatakar thikeyan et al. (18)	Case report series	1	1	0	48 months	48 months	1: BOT dermoid cyst	1: BOT	1: removed cyst	15 min	0	1	0	0	0	<10 ml	1	0	0	2 days	0	0

*n, numbers; SD, standard deviation; NGT, nasogastric tube; BOT, base of tongue; LTGDC, lingual thyroglossal duct cyst; OSAS, obstructive sleep apnea syndrome; TBR, tongue base resection; TORS, transoral robotic surgery; FAMM, facial artery muscolomucosal flap*.

The most frequent pathology observed was obstructive sleep apnea syndrome (OSAS): 20 subjects were treated with lingual tonsillectomy, 2 subjects were treated with lingual tonsillectomy plus epiglottoplastic, and 1 subject was treated with lingual tonsillectomy plus epiglottoplastic plus adenotonsillectomy (13). Five subjects underwent lingual tonsillectomy for non-OSAS diseases (7). The second most performed surgery was laryngeal cleft repair treating 18 subjects (1, 8, 9, 12). The third most common intervention (7 subjects) was the exeresis of lingual thyroglossal duct cyst (6, 11, 15, 17). Neoplastic pathology was treated in six subjects (8, 21%), four of these with malignant disease of the base-tongue: three sarcomas (4, 5, 14) and one minor salivary gland tumor (15). The remaining two subjects were affected by a benign pathology: one hamartoma (12) of the base tongue and the other neurofibroma of the larynx (16). Lymphatic malformation (12) was identified in 4 subjects, one in the base-tongue and three in the hypopharynx-larynx site. The numbers and anatomical distribution relating to the rest of the TORS procedures are shown in Table 3.

**Table 3:** Diseases treated and anatomical localization of TORS procedures.

**Diseases**	***N* of subjects**
Laryngeal cleft	18
Oroph. sarcoma	3
LTGDC	7
OSAS	23
Lingual tonsil non OSAS	5
Lingual thyroid	1
Minor salivary gland tumor	1
Lingual bronchogenic cyst	1
Glottic stenosis	1
Vocal cord paralysis	1
Lingual hamartoma	1
Lymphatic malformation	4
Saccular cyst	2
Dermoid cyst	1
Vallecular cyst	1
Pharingeal-esopphagous strictures	2
Neurofibroma	1
**Anatomical sites**	***N*** **of subjects**
Oropharynx	45
Larynx	23
Pharynx/hypopharynx/esophagus	5

*LTGDC, lingual thyroglossal duct cyst; OSAS, obstructive sleep apnea syndrome*.

There was only one intra-operative complication (1.36%): a small buccal laceration that required a suture in a laryngeal cleft repair surgery (8). There were a total of nine postoperative complications (12.32%) worthy of note, including four pneumonia (7, 12, 19), one of which with septic shock (12), 4 bleedings (7, 10, 15), and 1 edema of the tongue, which did not allow correct oral nutrition (9). There were four conversions (5.47%): three for oral exposure difficulties (1) and one subject was affected by posterior glottic stenosis. For the latter subject, a robotic-assisted posterior cricoid split with cartilage graft placement was attempted [but required conversion to an open surgical technique (9)].

Only nine studies reported operating times but due to the heterogeneity of the pathologies treated, we did not perform any statistical analysis.

On intraoperative blood losses, only eight studies reported these data (5, 7, 8, 10–12, 15, 18), and in all cases, it was <25 ml, except one subject <50 ml (15).

The results of the NICE methodology checklist for quality assessment for the case series are shown in Table 4. Data quality of 13 studies (81.25%) was classified as moderate and 3 as high (18.75%).

**Table 4:** National Institute for Health and Care Excellence (NICE) methodology checklist for quality assessment of case series.

**References**	**Question 1**	**Question 2**	**Question 3**	**Question 4**	**Question 5**	**Question 6**	**Question 7**	**Question 8**	**Total**
Rahbar et al. (1)	0	1	0	1	0	1	0	0	3
Kokot et al. (4)	0	1	0	1	0	1	1	1	5
Wine et al. (5)	0	1	0	1	0	0	1	1	4
Kayhan et al. (6)	0	1	0	1	0	0	1	1	4
Leonardis et al. (7)	0	1	1	1	0	1	1	1	6
Leonardis et al. (8)	0	1	1	1	0	1	1	1	6
Ferrell et al. (9)	0	1	1	0	0	1	1	0	4
Thottam et al. (10)	0	1	1	1	0	1	1	1	6
Carroll et al. (11)	0	1	0	1	0	0	1	1	4
Zdanski et al. (12)	0	1	1	1	0	0	1	1	5
Montevecchi et al. (13)	0	1	0	1	0	0	1	1	4
Canevari et al. (14)	0	1	0	1	0	0	1	1	4
Kayhan et al. (15)	0	1	1	1	0	0	1	1	5
Arnold et al. (16)	0	1	0	1	0	0	1	1	4
Turhan et al. (17)	0	1	0	1	0	0	1	1	4
Venkatakarthikeyan et al. (18)	0	1	0	0	0	0	1	1	3

*Low evidence quality: 0–2*.

*Moderate evidence quality: 3–5.*

*High evidence quality: 6–8.*

*Questions 1–8:*

*1. Case series collected in more than one center, i.e., multi-center study.*

*2. Is the hypothesis/aim/objective of the study clearly described?*

*3. Are the inclusion and exclusion criteria (case definition) clearly reported?*

*4. Is there a clear definition of the outcomes reported?*

*5. Were data collected prospectively?*

*6. Is there an explicit statement that subjects were recruited consecutively?*

*7. Are the main findings of the study clearly described?*

*8. Are outcomes stratified? (e.g., by disease stage, abnormal test results, patient characteristics)*.

## Discussion

The use of the robot in the pediatric transoral robotic procedure has been more consistent over the years. The first work on the application of robotics in the pediatric population was conducted by Rahbar et al. (1). Since then, several studies have been published regarding pediatric TORS. The first and only article reviewing the literature on this topic was published in 2015 by Erkul et al. (19). They analyzed studies concerning 41 subjects and the surgical procedures carried out. Since then, 32 new pediatric subjects have been reported in the literature, approximately doubling the number of patients to be analyzed; however, the number of subjects included is quite low and there are lots of missing data in the selected articles. These aspects limit this work and prevent us from drawing any definitive conclusion.

The most surgically treated site was the oropharynx, especially the base of the tongue (Table 3). In this anatomical location, the applications for surgery have become broader and there has been an increase over the years of cases of treated Lingual Thyroglossal Duct Cyst (LTGDC) and the use of TORS for the malignant neoplastic pathology. Until 2017, information is only available for the surgical repair procedures of the laryngeal cleft, posterior glottic stenosis, and idiopathic bilateral cord paralysis. In the following years, 2 saccular cysts, 3 lymphatic malformations (12), and a neurofibroma involving the parapharyngeal space (16) were successfully removed.

Four conversions (5.47%) are identified: 3 for oral exposure difficulties, all in the first study of the series (1). The other case of conversion was because of the inability to have a good visual of the subglottis and difficulty with the placement of robotic arms in a subject with posterior glottic stenosis, following an inhalational burn injury (9). The learning curve provides the authors with a plausible reason behind the lack of other conversions in the series, other than the ones already described, despite more complex surgical interventions over the years. Only a minor buccal laceration is described for intraoperative complications.

We consider the data relating to post-operative bleeding and tracheostomies, to be interesting for its variations. Four among the 73 subjects (5.47%) were reported with postoperative bleeding. Hay et al. (20) reported in adult subjects an incidence of postoperative bleeding after TORS procedures of 16% and 6% among the subjects back to the operating rooms to manage the complication. In other large retrospective TORS series on adult subjects, the risk of postoperative hemorrhage ranged from 7 to 22% (21). Due to the low incidence of bleeding in pediatric subjects, we agree with Canevari et al. (14) that tracheostomy can only be justified in selected cases and no intra- or post-operative tracheostomies were reported in the selected studies. Only 11 subjects were subjected to this procedure due to a difficulty in breathing but before the surgery.

In terms of intraoperative blood loss, we have not noticed considerable data on the amount of milliliters. We hypothesize that it would have been of interest to conduct a comparison based on incidence percentages of the intraoperative blood losses. The comparison could have been made on data with adults or with non-robotic transoral procedures. Unfortunately, among the 16 studies of the series reported, only 8 had the available data, not enough to conduct a statistically relevant comparison; however, it must be taken into account that the experience on pediatric TORS procedures is derived from the experience of adult patients, limiting any serious comparison of data regarding intra- and post-operative complications.

The endotracheal tube does interfere with a good visualization (14) of the oropharynx, and, despite some surgeons prefer trans-nasal intubation to treat the base of the tongue (15), others reported that both oral or nasal intubation did not obstruct the necessary view of the oropharyngeal anatomy (8). In another significant study on subjects operated at the base of the tongue (15), these authors argue that compared to adult subjects, in pediatric subjects the limits are because the size of airways in children is smaller, mouth opening is inefficient, have large retractors and the available instruments today reduces exposition leading to incompatibility of robotic arms. These authors used different types of mouth opening devices, such as the Davis-Meyer mouth gag, Davis-Boyle mouth gag, Dingman retractor, McIvor retractor, and Feyh-Kastenbauer retractor, depending on age and mouth structure of the child; however, a laryngeal saccular cyst was removed in a 14-day-old patient successfully and with no complication.

The use of the Da Vinci Xi system was made in four different studies and four consecutive years from 2017 until today (15–18). Its application has remained marginal, in part due to the non-FDA approval for TORS procedures, and in part because of the technical peculiarities that limit its applications for certain TORS procedures (22).

We agree with Zdanski et al. (12) that the plausible key elements that can lead to further evolution of TORS procedures in pediatric subjects are the following: securing the airway tract with the appropriate laser-safe endotracheal or tracheostomy tubes; identifying the appropriate exposure; surgical access with robotic arms for unrestricted mobility; the critical role of the bedside surgeon, in protecting the airway, the patient, and assisting the robotic surgeon. It will be necessary in the future to design appropriate instruments for pediatric airway TORS surgeries, without converting the general or urological instruments available today. The Da Vinci Robotic System was built to treat general and urological pathologies, and, nowadays, its instrumentation is adapted to TORS procedures (22). For that reason, most of the time patient selection is tailored to what can be done with the available technology and not vice versa.

## Conclusion

The feasibility of the pediatric TORS procedures has been demonstrated up to the laryngeal region, with good outcomes in terms of successful surgeries. In addition to the evolution of the instruments, we hypothesize that surgeons who already currently practice this type of surgery in adult subjects can also upgrade their training and experience in a pediatric patient.

In relation to the low intraoperative blood loss, the lack of major intra- and post-operative complications, the low percentage of the minor ones, and the low percentage of conversions in open procedures, we can consider the TORS procedures in pediatric subjects safe and feasible.

## Data Availability Statement

The original contributions presented in the study are included in the article/supplementary material, further inquiries can be directed to the corresponding author/s.

## Author Contributions

MV and GF: study conception and design. MV, GF, and GB: data acquisition. MV, GF, ID, and LB: analysis and data interpretation. MV, GF, and LB: drafting of the manuscript. LB: critical revision of the manuscript. All authors contributed to the article and approved the submitted version.

## Conflict of Interest

The authors declare that the research was conducted in the absence of any commercial or financial relationships that could be construed as a potential conflict of interest.

## Publisher's Note

All claims expressed in this article are solely those of the authors and do not necessarily represent those of their affiliated organizations, or those of the publisher, the editors and the reviewers. Any product that may be evaluated in this article, or claim that may be made by its manufacturer, is not guaranteed or endorsed by the publisher.
